# Iron Oxide Powders Containing Arsenic from Water Treatment Processes Mixed with Cement as Environmental and Structural Solution

**DOI:** 10.3390/ma18030582

**Published:** 2025-01-27

**Authors:** Henry A. Colorado, Jeiser Rendón Giraldo, Manuela Montoya, Mauricio Correa, Mery Cecilia Gómez Marroquín, Sergio Neves Monteiro

**Affiliations:** 1 CCComposites Laboratory, School of Engineering, Universidad de Antioquia—UdeA, Calle 70 No. 52-21, Medellin 050010, Colombia; grupoglima@udea.edu.co (J.R.G.); fc@uni.edu.pe (M.M.); 2 Grupo de Investigación y Laboratorio de Monitoreo Ambiental—GLIMA, Facultad de Ingeniería, Universidad de Antioquia—UdeA, Calle 70 No. 52-21, Medellin 050010, Colombia; scoms@ime.eb.br; 3 National University of Engineering, 210 Túpac Amaru Ave, Rímac, Lima 15333, Peru; merycgm@gmail.com; 4 Departamento de Ciência dos Materiais, Instituto Militar de Engenharia, Rio de Janeiro 22290-270, Brazil; snevesmonteiro@gmail.com

**Keywords:** iron oxide powders stabilizations, arsenic management, cement paste, water treatment, CO_2_ emissions reduction

## Abstract

This study explores the stabilization and utilization of hazardous waste (HW) derived from iron oxide powders containing arsenic, a byproduct of a water purification process. Cement paste samples were prepared with varying waste content (0.0%, 2.5%, 10% and 20% by weight) through mechanical mixing of all the components. Utilizing this waste offers two key environmental benefits: first, it addresses the issue of large-scale waste production globally by providing a method for its stabilization; second, it reduces cement consumption in concrete by serving as an admixture and filler, thereby lowering the cement industry’s significant CO_2_ emissions. After 28 days, compressive strength and density tests were conducted, and the microstructure was examined using scanning electron microscopy and X-ray diffraction. The results demonstrated compressive strengths exceeding 20 MPa, with the presence of calcite, portlandite, and ettringite phases in the samples. Additionally, Weibull statistics were conducted over a wide number of samples per composition in order to account for the variability of the compression properties, which can be important for deciding the applications. The results showed that the prepared formulations can be used in structural applications such as walls, infrastructure, sidewalks, and soil stabilization.

## 1. Introduction

Access to clean, fresh water is a fundamental human right and is essential for subsistence. However, in recent decades, it has become a resource that is increasingly difficult to access due to a combination of factors that include overpopulation, deforestation, intensive agriculture, pollution, poor management of this resource and global warming [1,2]. Currently, one in four people in the world do not have clean drinking water access, and this is a major worldwide problem that requires priority attention [3].

In some countries, there is a serious problem with the lack of fresh water suitable for human consumption, especially in African countries with few water resources that also share a main source of water with other countries [4,5]. In addition to these, it is common to find water sources contaminated with heavy metals such as arsenic, lead, cadmium and chromium, which are not biodegradable materials, as is the case in India, Bangladesh and other countries [6], where thousands of people drink waters contaminated with hazardous metals [7]. Moreover, it is common to find foods from the daily market contaminated with these metals. This occurs especially due to the poor management of wastewater, irrigation of crops with water contaminated, intensive use of pesticides and fertilizers and traces of these compounds in feed for farm animals [8,9,10].

Furthermore, large-scale pollution in water is a major issue worldwide, with loss of biodiversity at a rate that is almost 1000 times higher than that of nature itself, depriving future generations of an invaluable inheritance and causing an unprecedented extinction, all of this with immense consequences for the public health and sustainability of the planet [11]. Notably, the excessive growth of industry and residential areas is immensely altering ecosystems, affecting both aquatic and terrestrial species, altering the climate, all of this obviously bringing up consequences for people [12].

Approximately 80% of wastewater in the world falls directly into water sources without any type of treatment, and this is causing an immense amount of illnesses and deaths around the world, especially among the child population [13]. Different strategies have been used for the recovery and reuse of wastewater, some as old as activated sludge and others as new as the use of nanoparticles. Although different treatments have been implemented for several decades to achieve adequate discharge of wastewater into the environment, procedures have also been used in recent years to reuse wastewater [14,15]. Today, water recovery is the basis of solutions to reduce a problem that seems far from a real solution due to the scale and the lack of real interest of governments in the world, which can be reflected in the limitation in the local and international regulations for the sustainability of the planet [16].

Arsenic contamination in water sources is a pressing issue for many countries, including Bangladesh, where it has escalated into a public health crisis, often referred to as the largest mass poisoning in history [17,18,19]. This problem is widespread and affects numerous regions globally. Prolonged exposure to arsenic in drinking water is associated with an increased risk of cancers, including those of the skin, lungs, bladder, and kidneys. Additionally, it has been linked to various non-cancerous health conditions, such as diabetes, peripheral neuropathy and cardiovascular diseases [20]. Recognizing these dangers, some countries, including the United States and Canada, have tightened regulations by reducing the maximum permissible arsenic level in drinking water from 50 to 10 μg/L [21]. However, many developing countries lack sufficient regulations or have no laws governing the use of arsenic in products like pesticides, which could lead to widespread contamination [22]. Various techniques have been developed to remove arsenic (including As (III) and As (V)) from water or to treat processes with elevated arsenic levels. These methods include oxidation, phytoremediation, coagulation-flocculation, electrocoagulation, adsorption, ion exchange, electrokinetics and membrane technologies [23]. Among these, adsorption—commonly utilizing iron-based sorbents—often results in the generation of substantial amounts of arsenic-laden ceramic powder [24,25]. Disposing of this iron oxide-based byproduct poses significant challenges due to its arsenic content.

Cement and concrete, as the most widespread manmade materials, have been used for many years in combination with different byproduct materials and wastes [26], which can work as admixtures [27], fillers [28], reinforcements [29] or in any other functionality. Due to the amounts of concrete demanded worldwide, where the global annual consumption of concrete is close to 30 billion tons [30], the use of wastes into the concrete formulations is important to this industry, as the amount of cement or aggregates available is due to its high demand, costs and sometimes scarcity for specific materials and formulations [31]. Thus, concrete made with cement and fly ash is a success [32] for the sector, as this byproduct from the steelmaking industry has pozzolanic activity and also contributes to the hydration and mechanical properties of Portland cement [33]. Concrete research and development include products such as brick waste [34], construction and demolition waste [35], mine waste and tailings [36], natural fibers [37] and metallurgical waste from diverse processes [38], among many others. Therefore, the use of waste is very beneficial for this industry because of the properties, availability of raw materials and costs reduction [39] but very significant because concrete is responsible for about 8% of the total CO_2_ emissions [40], and by using waste, these numbers can be reduced, thus contributing to reducing the atmospheric pollution [41,42]. The most used method for introducing wastes in concrete, particularly hazardous wastes, is the solidification stabilization (SS) method [43].

Cement and concrete, on the other hand, are materials that can be naturally exposed to high temperatures by fire or by their use [44,45]. Portland cement when exposed to fire has a problem known as spalling, critical damage under fire that can even contribute to expanding the fire in a building [46]. Spalling often results in the loss of concrete cover, which diminishes the thermal insulation protecting the reinforcement. This exposes the confined concrete core to greater thermal degradation and increases the risk of high-temperature exposure for the reinforcement steel.

The material response under these conditions is quite complex and undergoes very extreme chemical and mechanical changes [39] that include cracking and creeping [47], creating new high temperature phases, but also producing micro- and macrocracking, shrinkage and voids [48]. Also, macro damages are produced, which included cracks, destruction of decoration and surface damages. There is a lack of data of cement with different waste materials studied under these conditions.

This study aims to investigate the valorization and stabilization of arsenic-containing iron oxide compounds in white Portland cement (WPC) paste, derived from the treatment of arsenic-contaminated water, with emphasis on the evaluation of the mechanical properties of a comparable mixture. The technology of solidification/stabilization (S/S) has been proven to successfully limit the mobility of hazardous species and atoms in concrete and other cementitious materials [48], and a study was even conducted to prove it in stabilizing arsenic wastes in cement [48] using the toxicity characteristic leaching procedure (TCLP). This study proves arsenic leaching into water when using Portland cement and having acceptable limits and following the EPA regulations. Therefore, the proposed research is focused in proving the feasibility of using these materials in the construction sector, which can really drive the solution to use the waste. In future, further TCLP tests can be conducted upon more extreme conditions and over concrete mixes rather than cement, which is a need for a full scalability of the mixes.

Thus, this research aims to understand the relationship between cement paste incorporating arsenic and its use as a binder for structural applications. To achieve this, compressive strength and density tests were performed on various formulations, while the chemistry was analyzed using scanning electron microscopy for understanding the microstructure and X-ray diffraction for understanding the crystalline structures. Another objective of this study is to explore the issue of arsenic contamination, with potential applications in developing countries like Colombia, where regulations on arsenic in food and agriculture are lacking [22,49] and processes using or effectively recycling this type of waste are necessary to decrease pollution [50].

## 2. Materials and Methods

The preparation process first involved mechanically mixing water and cement in a Hobart planetary mixer, followed by a gradual addition of waste material. A constant water–cement ratio (W/C) of 0.4 was maintained in all samples to ensure comparability. Formulations included waste contents of 0.0, 1.0, 2.5, 5.0, 10 and 20% by weight. Up to 40% by weight of waste was attempted, but these mixtures proved unsuccessful due to poor flow (not quantified in this research), inadequate water content and handling difficulties.

The samples were then cured at room temperature in sealed containers to minimize exposure to air and demolded after 28 days for testing. A detailed summary of the powders used in the sample formulations is provided in the table below (Table 1).

Compression tests were then performed using samples with dimensions of 20 mm diameter and 24 mm height on a Shimadzu Autograph universal testing machine at a head speed of 1 mm/min on six samples for each cement and waste formulation.

X-ray diffraction (XRD) analysis was then performed using an X’Pert PRO diffractometer with Cu Kα radiation (wavelength 1.5406 Å) on all raw and manufactured waste samples in powder form. The XRD scans covered a 2θ range of 5–70° with a step size of 0.02°.

Microstructural observations of the cured cement samples were carried out using a JEOL JSM–6490 scanning electron microscope (SEM). Density tests were performed on six samples by calculating the mass–volume ratio based on their measured weights and dimensions. These compressive strength and density results were presented as mean values with their respective standard deviations.

The temperature exposure tests were then carried out in a conventional oven without atmospheric control at 500 and 800 °C for 1 h. The samples were heated and cooled with temperature ramps of approximately 1 °C/min. To analyze the data from the mechanical compression tests, the Weibull statistic was used to take into account the variability of the results, with 20 samples per formulation.

Finally, the number of samples was selected to be 20 samples per formulation fabricated—more than 10, the minimum number of samples for Weibull statistics in ceramics and fragile materials [51]. Certainly, the more samples, the better the analysis, but this research was limited in making more samples because of the amount of waste provided.

## 3. Results

**Figure 1:**
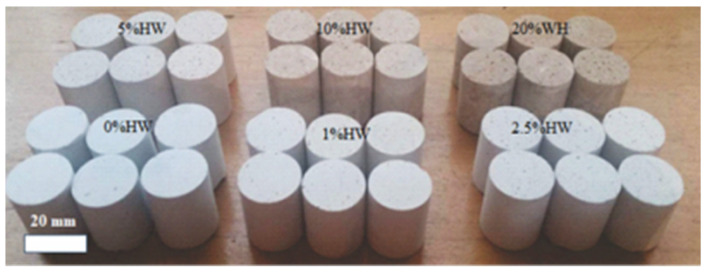
Typical samples fabricated in this research with different waste contents for compression and other tests.

Long-term exposure to inorganic arsenic, mainly through drinking water and food, can lead to chronic arsenic poisoning, as has been demonstrated in several publications [52,53]. Samples made for density and compression tests fabricated in this research are shown in Figure 1.

**Figure 2:**
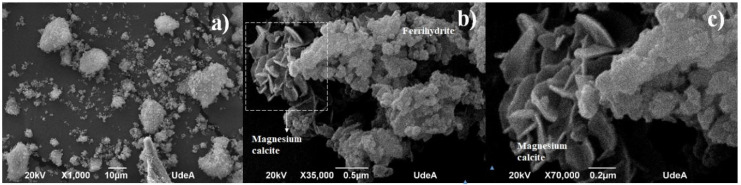
Raw powder used as an admixture for making cementitious samples at three different magnifications: (**a**) 1000×, (**b**) 35,000× and (**c**) 70,000×.

The morphology of the waste powder is depicted in Figure 2, captured using SEM. The powder is very fine and has a complex shape, primarily consisting of numerous agglomerated particles, as shown in Figure 2a. At higher magnification (Figure 2b), phases of magnesium calcite and ferrihydrite are visible, which were later confirmed through XRD analysis. The ferrihydrite phase exhibits an agglomerated particle structure, with some individual particles reaching the nanoscale. These particles are round in shape. In contrast, magnesium calcite displays a complex, branched structure, with some micro-plates forming a flower-like arrangement.

**Figure 3:**
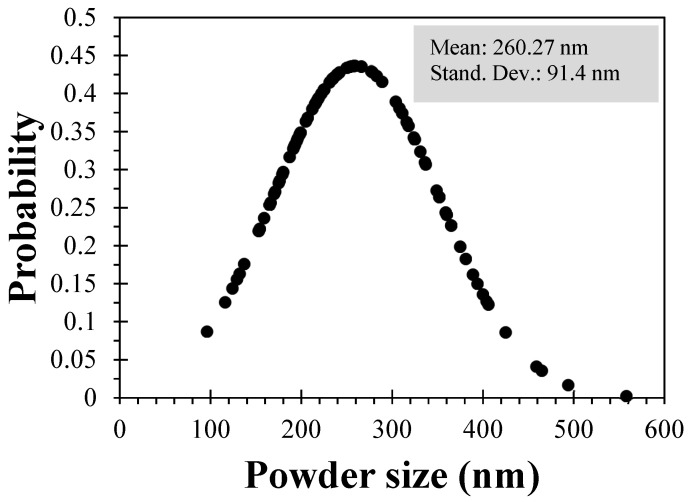
Particle distribution of the powder residue.

Figure 3 shows the particle size distribution of the powder residue, with an average size of 260 nm (2.6 µm), which, due to its size, can favor the cementitious matrix, either by filling pores or achieving chemical interactions with the cement.

**Figure 4:**
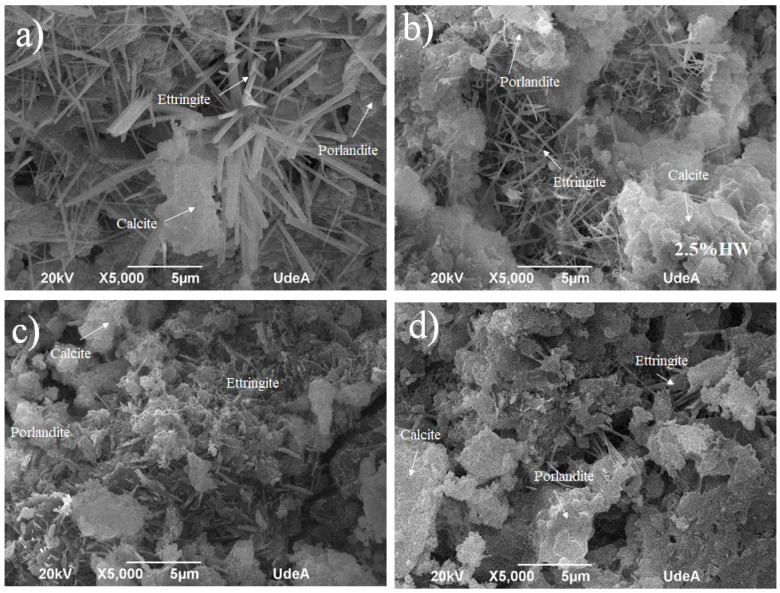
SEM images taken of the formulations made of iron oxide containing arsenic waste and WPC at concentrations of (**a**) 0.0 HW%, (**b**) 2.5 HW%, (**c**) 10 HW% and (**d**) 20 HW%.

Figure 4 presents a summary of the SEM images of the WPC samples containing waste taken after the samples were demolded from 28 days of curing. The images focus on fractured surfaces and pores, which provided favorable conditions for the nucleation of ettringite. This phase was identified in all the formulations. Samples with 0.0 and 2.5 HW% waste exhibited a significant presence of ettringite, as shown in Figure 4a,b by long needle-like microstructures. However, as the iron oxide-based waste containing arsenic increased, the quantity of the ettringite phase diminished to short and fewer structures, as seen in Figure 4c,d. The irregular formations visible in these latter images are identified as calcium–silicate–hydrate (C–S–H).

**Figure 5:**
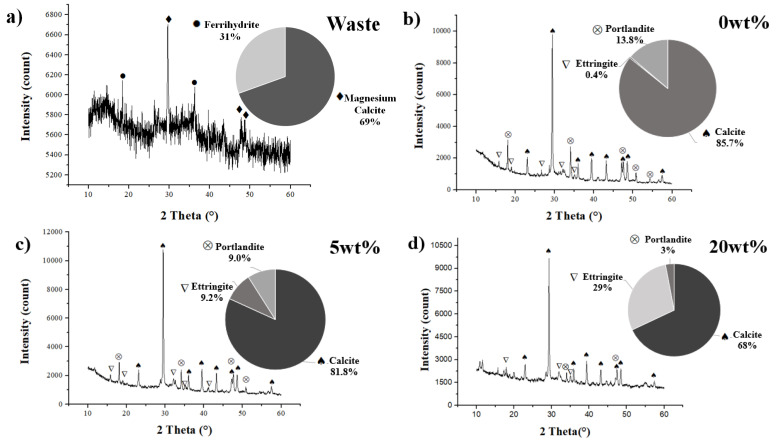
XRD with a Rietveld analysis of (**a**) raw waste powder, (**b**) 0.0 HW%, (**c**) 5 HW% and (**d**) 20 HW%.

Figure 5 provides a summary of XRD data for the raw waste material taking over the fabricated samples. Figure 5a displays the XRD pattern of the waste powder, which is iron oxide-based with arsenic. The analysis identified the presence of magnesium calcite (CaCO_3_) and ferrihydrite ((Fe^3^⁺)_2_O_3_·0.5H_2_O) phases. Figure 5b–d present the XRD spectra for WPC samples containing 0.0, 5.0 and 20 HW% waste, respectively. In all cases, typical cement phases were observed, including calcite, portlandite and ettringite, which certainly contribute to encapsulate and stabilize the arsenic contents [54]. However, an increase in waste content corresponded to a reduction in the ettringite phase and, as it will be presented later, a reduction in the compressive strength due to several factors that include less cementitious phases. The XRD of the waste containing arsenic is much less crystalline than the XRD of the formulations with cement, which indicates a completely different material with expected different properties, including different impacts on the environment.

**Figure 6:**
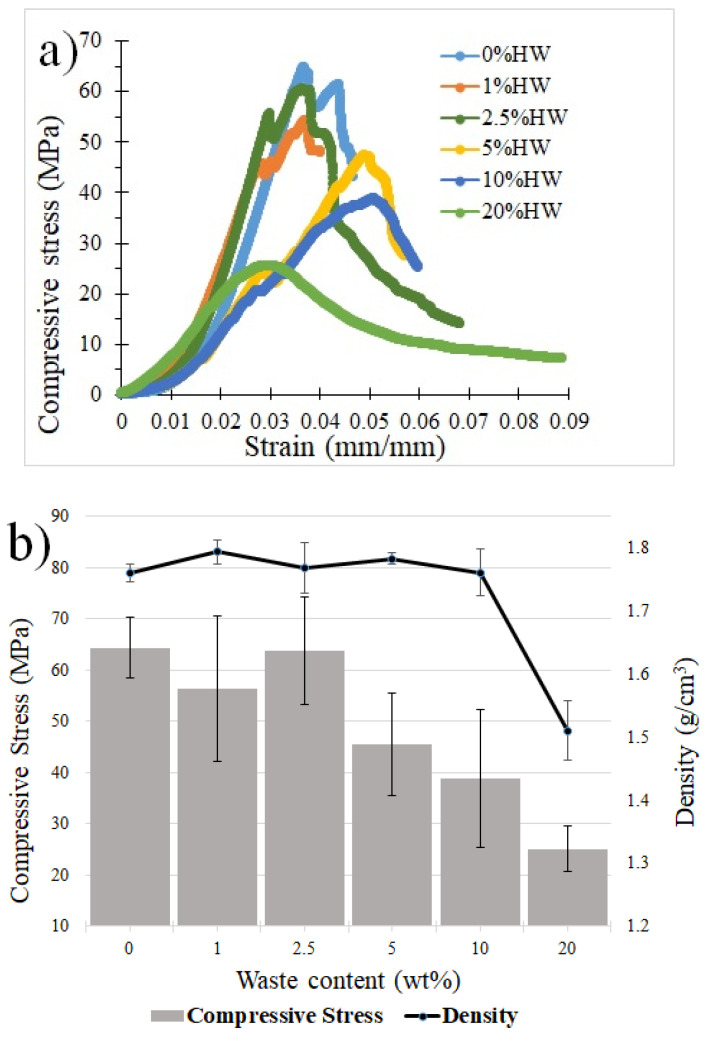
(**a**) Compressive strength results, and (**b**) density tests data for the differently made formulations of WPC with iron oxide containing arsenic.

Figure 6 shows the density and compression tests of the formulations. The density is quite stable up to 10 HW% of waste, with a value around of 1.8 g/cm^3^, and has a significant drop when double the waste is added, 20 HW%, to around 1.5 g/cm^3^, which is related to the increase in the voids generated with the high loading of the waste. Compression strength keeps the highest values (near 60 MPa) up to 2.5 HW% of waste added, and then, with 5, 10 and 20 HW%, the values decrease to near 45, 39 and 25 MPa. This is mainly expected because of two factors: the decrease in density and the waste particle agglomeration as waste loading increases. The density decrease can explain only part of this behavior, as this remains almost constant up to 10 HW% of waste, followed by a significant decrease with more waste added. Therefore, it is the agglomeration increase and the corresponding poor bonding waste to the cement matrix mainly responsible for the deterioration of the compressive strength. It is important to consider another possible deterioration that the cement matrix may suffer due to the reaction of iron oxide powders containing arsenic with oxygen [55].

**Figure 7:**
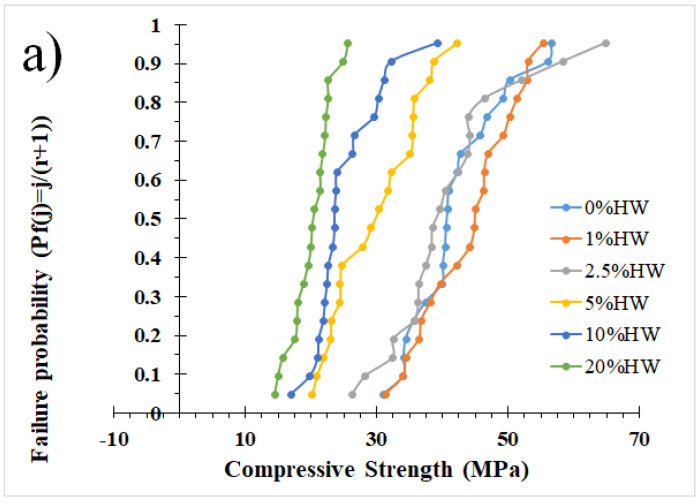

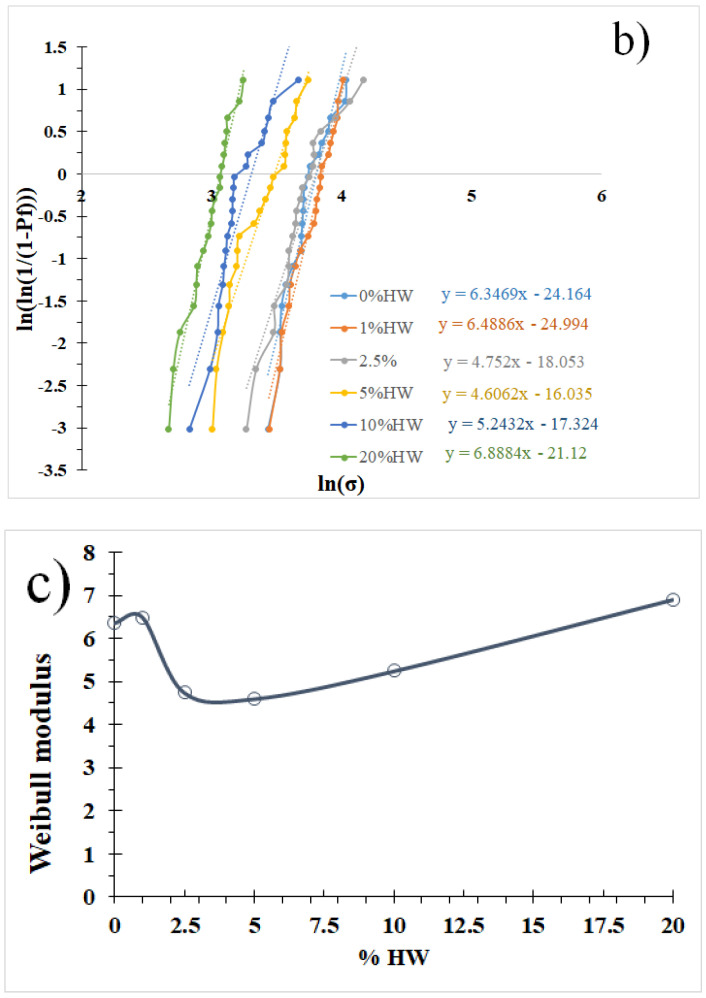
Weibull statistics data for different formulations of samples of WPC and waste containing arsenic. (**a**) Failure probability. (**b**) Weibull fit. (**c**) Weibull modulus.

Figure 7 shows the Weibull distribution statistics. Twenty samples were tested per formulation, as shown in Figure 7a, for 0 HW% waste compression values ranging from 31 to 56.6 MPa, for 1 HW% from 31 to 55.4 MPa, for 2.5 HW% from 26.2 to 65 MPa, for 5 HW% from 20 to 42.3 MPa, for 10 HW% from 17 to 39 MPa and for 20 HW% from 14 to 25.6 MPa. The Weibull fit shown in Figure 7b and summarized in Figure 7c just for the Weibull modulus showed that 1 HW% of waste increased the modulus from 6.3 to 6.5, which decreased the property variability. This improvement may be associated with the fact that the waste particles fill the gaps and therefore reduce the defects in the mixture, an issue that has already been highlighted in other publications, showing how the use of nanoparticles incorporated into the cement mixture can lead to better cement performance by increasing its strength (between 5 and 25%) [56].

Additions of 2.5 and 5 HW% of waste decreased the modulus notoriously, which means they increased the compressive strength variability, supporting the agglomeration and property deterioration with the waste loading from Figure 6, which is closely related to the increase in particle agglomeration, micro- and nanopores and even micro- or nanocracks. Thus, the further addition of waste to the contents investigated (5.0% HW) causes a significant strength decrease (see Figure 6). The reduction and control of these defects can lead to higher values of the Weibull modulus, which can be done by optimizing different processing parameters of the mix, such as mixing and curing [57]. Vacuum technologies [57] can help but increase the costs and complexity of the fabrication. The higher the defects and variability in the properties, the lower the Weibull modulus, with more possibility of macrodefects to appear.

Better control of the defects can produce an increase in the modulus (see Figure 7c), which is translated into less property variation, probably associated with the waste dominating the compression behavior. It is worth noting that, even with 20 HW% waste content, the compressive strength values remained acceptable for applications that do not require high strength, such as sidewalks, decorative elements or as a stabilizing material for hazardous waste, where microdefects and variability, as accounted by the statistical analysis, such as those shown in here, can be acceptable.

**Figure 8:**
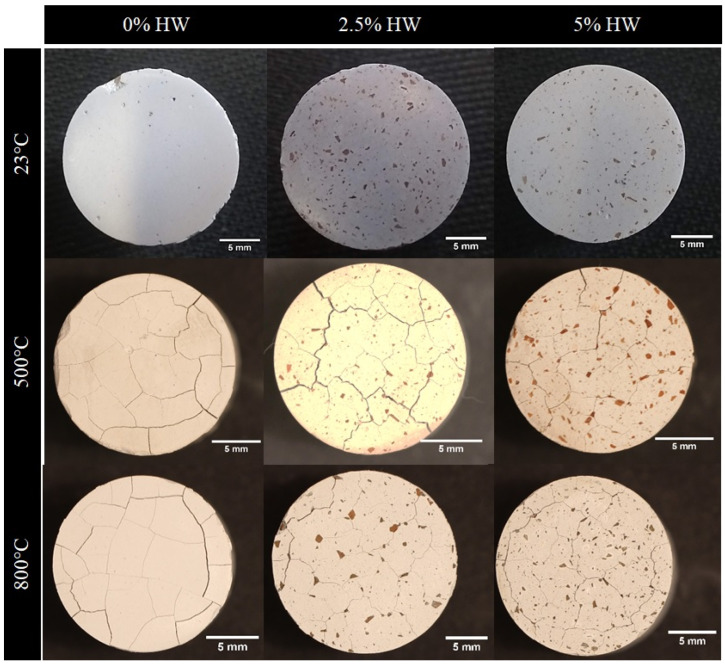
Thermal damage evolution after exposure of the samples to different temperatures in a furnace open to the air for 1 h.

**Figure 9:**
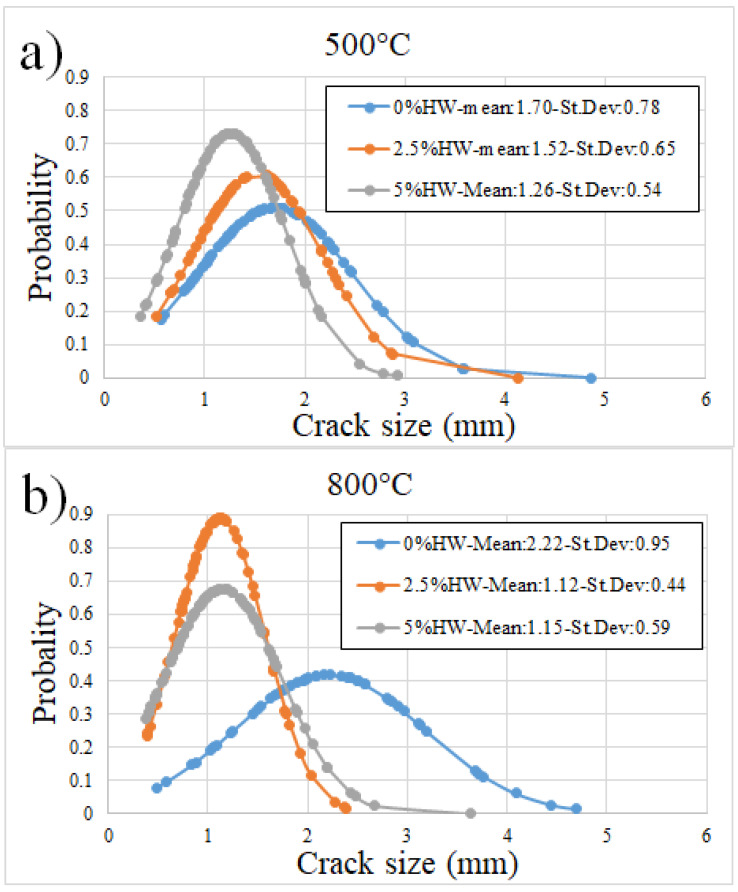
Crack length distribution for samples after being exposed to temperatures of (**a**) 500 °C and (**b**) 800 °C during 1 h in air atmosphere.

Figure 8 shows the cross-section areas of the samples upon being exposed to 500 and 800 °C for 1 h in air atmosphere for samples with 0, 2.5 and 5 HW% of waste. As a reference, the sample with 0 HW% of waste is presented as made at room temperature. In general, these two temperatures cause significant damage, although the samples were kept stable in shape. A quantification of these damages is presented in Figure 9a for samples exposed to 500 °C and Figure 9b for samples exposed to 800 °C. At room temperature, for the samples as made, there are no cracks. Figure 9a shows that, as the waste loading increases from 0 to 2.5 and 5% of waste, the crack size is diminished from 1.7 to 1.52 and 1.26 mm. This is a good result and is probably associated with the particles stopping cracks. At 800 °C is a significant temperature for cement, causing damages and interdiffusion among different phases, additives and materials in the mix. Figure 9b shows that, at 800 °C, the waste has a more significant effect on the crack size, with a reduction from 2.22 to 1.12 and 1.15 mm for samples with 0, 2.5 and 5 HW% of waste, respectively.

The data from XRD did not reveal a strong influence of the waste on the cement chemistry, as the found phases were associated with cement when compared to the waste powder (see Figure 5a). However, Figure 8 shows macroscopic changes introduced by both the waste additions and the temperature damage. Waste agglomeration of about 1 mm or less appeared in samples 2.5% HW and 5.0% HW, which certainly is a defect that causes issues in the mechanical properties, such as a decrease in strength with the increase in waste content. Moreover, this waste combined with temperature showed a profound effect on the cracking after thermal exposure of the samples, with cracks near 5 mm in length (see Figure 9). Therefore, reducing these microdefects can lead the material to important improvements, which must be further investigated via processing (for instance, mixing and thermal technologies) and materials modification (additives and phase modification).

## 4. Discussion

The results of this research show a viable way to manage arsenic-contaminated water. The use of arsenic-bearing iron ores in concrete basically only needs logistics costs of transportation if used directly and in the powder sizes of the raw materials as used in this research, because they do not require further processing [58]. Other strategies like recovering iron are not related to this research and are costly [58], which need more research to make a solution possible. A realistic study involving costs requires a life cycle assessment evaluation of the proof of a large-scale implementation.

Regarding the properties of the developed formulations, the compressive strength values are competitive for many structural applications, including housing, side roads, barriers and others. This can be supported by the standard ASTM C270-24 (Standard Specification for Mortar for Unit Masonry) [59,60,61], which provides the basis for specifying cement-lime mortars from classification, property and applications. All types have minimum averages over 2.4 MPa (M: 17.2 MPa, S: 12.4 MPa, N: 5.2 MPa and O: 2.4 MPa). The building segments for N are load-bearing walls and parapet walls; for O, non-load bearing walls and tuckpointing; and for S, foundation walls, retaining walls, pavements, manholes, sewers, walks and patios. M can be also used for load-bearing walls, foundation walls and the S applications. Since all the results in this research showed a mean compressive strength of 20 MPa in the worst case for the mix, in principle, upon optimization, the formulation can be used in all these applications, especially when mixed with aggregate that can improve the properties.

It is clear that data from this research can be improved by using additives and a combination of reinforcements, such as ceramic fibers, particularly for reducing the high temperature cracking found after the sample exposure at 500 and 800 °C.

In the Amazons, it has been reported that some sources are contaminated with arsenic and other hazardous metals derived mainly from gold mine exploitation [62], which requires further study and government treatment for water sources near human consumption. This same situation can be seen in other South American countries, such as Colombia, where the Sutará River suffers from similar conditions, Western Amazonia in Perú, Altiplano-Puna in Argentina, Chile and Bolivia, and it is a situation that can be replicated throughout the South American continent, where around 14 countries suffer from similar problems [63,64,65].

## 5. Conclusions

The inclusion of iron oxide-based waste containing arsenic demonstrates promising results as an admixture or filler in cementitious materials. Even at concentrations of up to 20 HW%, the waste produced a composite material with acceptable strength, likely due to its fine particle size and iron oxide composition, which are comparable to some components of conventional cement. This indicates that stabilizing such waste in cement is an effective and practical solution for managing this contaminant. Furthermore, this approach could be applied in other countries facing similar waste management challenges. The following conclusions were obtained:

- The developed formulations are a solution for using the waste in structural applications such as walls, infrastructure, sidewalks and soil stabilization. This is possible due to the competitive values obtained in the compressive results, ranging from 60 MPa mean compressive strength in the best cases to 20 MPa in the worst case, from low to high waste concentrations.
- As expected, as the amount of added waste increased in the cementitious formulation, the compressive strength decreased, which can be associated with less binder material (Portland cement) and more defects (voids, waste particle agglomeration and poorest waste to cement bonding).
- The Weibull statistics showed that the variability of the tests was among the expected values of the Weibull modulus, about 6. This result can be improved by decreasing the defects (voids, waste particle agglomeration and poorest waste to cement bonding) via processing the parameters and additives.
- Density values were very consistent up to 10 HW% of waste, which suggests a limitation for high strength and density over this value due to a higher concentration of defects.
- Pollution decrease is the main contribution of this research. Further studies may focus on studying how to guarantee long-term stability in the water of these materials, avoiding degradation that can release arsenic into the soil. This test can be performed by accelerating the water damage effects by doing the tests at higher temperatures and at different pH conditions. Suggested temperatures can be 30, 50, 70, 100 and 150 °C for 1 and 4 weeks. The pH can be introduced in combination with the temperature variation from acidic to alkaline, for instance, running in conditions from 2 to 13.

## Figures and Tables

**Table 1:** Composition of the formulations fabricated in this investigation from waste containing arsenic and WPC.

Sample	Waste (HW%)	WPC (HW%)
0.0 HW%	0.0	100
1.0 HW%	1.0	99
2.5 HW%	2.5	97.5
5.0 HW%	5.0	95
10 HW%	10	90
20 HW%	20	80

## Data Availability

The raw data supporting the conclusions of this article will be made available by the authors on request.
